# Sensor Network Attack Synthesis against Fault Diagnosis of Discrete Event Systems †

**DOI:** 10.3390/s24144445

**Published:** 2024-07-09

**Authors:** Tenglong Kang, Yifan Hou, Ding Liu

**Affiliations:** 1 School of Electro-Mechanical Engineering, Xidian University, Xi’an 710071, China; tlkang@stu.xidian.edu.cn (T.K.); yfhou@xidian.edu.cn (Y.H.); 2 Department of Electrical and Electronic Engineering, University of Cagliari, 09123 Cagliari, Italy

**Keywords:** fault diagnosis, sensor network attack, discrete event systems

## Abstract

This paper investigates the problem of synthesizing network attacks against fault diagnosis in the context of discrete event systems (DESs). It is assumed that the sensor observations sent to the operator that monitors a system are tampered with by an active attacker. We first formulate the process of online fault diagnosis under attack. Then, from the attack viewpoint, we define a sensor network attacker as successful if it can degrade the fault diagnosis in the case of maintaining itself as undiscovered by the operator. To verify such an attacker, an information structure called a joint diagnoser (JD) is proposed, which describes all possible attacks in a given attack scenario. Based on the refined JD, i.e., stealthy joint diagnoser (SJD), we present an algorithmic procedure for synthesizing a successful attacker if it exists.

## 1. Introduction

With the advancement in computation and communication technologies, cyber–physical systems (CPSs) have emerged as the new generation of engineering systems and computation devices embedded within physical dynamics [1,2,3,4,5]. Examples of CPSs include sensor networks, networked computer systems, autonomous vehicles, etc. Usually, the undesired behavior of CPS may be caused by the occurrence of component faults. Hence, we wish that a fault can be diagnosed after its occurrence based on data collected by sensors. In a CPS, the transmission of data information relies on communication networks, which may introduce vulnerabilities to cyber-attacks.

In this paper, we study the behavior of a CPS within the framework of discrete event systems (DESs) [6]. One significant advantage of the DES approach to fault diagnosis is that it is a model-based approach. Therefore, DES models naturally capture faults as abrupt events in systems, which facilitates the analysis of faulty behaviors of the system based on limited observations. Another motivation is that, unlike complex models or large data-driven models, DES modeling offers an opportunity to make efficient decisions over an abstract discrete decision space. Specifically, a DES is an event-driven model-based formalism that can provide a time-abstract model for complex systems.

There are two main approaches and tools available for the fault diagnosis of a DES, i.e., automata theory [7,8,9] and Petri nets [10,11,12,13]. A comprehensive survey can be found in [14]. Within the context of automata theory, the underlying structure of a DES is that of a finite state automaton. This will be formally defined in Section 3. Online fault diagnosis in a DES aims to perform model-based inferencing at runtime based on the current observations, and determines if a fault event has occurred or not in the past. In order to achieve this, a process called a diagnoser is proposed in the seminal work [7], which associates to each observed word of events a diagnosis state, such as “negative”, “positive”, or “uncertain”.

## 2. Related Work

The related literature in the context of robust fault diagnosis against disruptions in the communication channels includes, e.g., [15,16,17]. For a survey of the work in this domain, we refer to [17]. These works are concerned with the loss of observations, i.e., any event in a certain subset of the observable event set may become unobservable due to sensor failures or communication channel constraints. It is shown in [17] that, based on a general diagnosis framework that takes sensor failures into account, a procedure of online diagnosis can be presented. However, such disruptions are not necessarily caused by active attacks.

**Figure 1:**
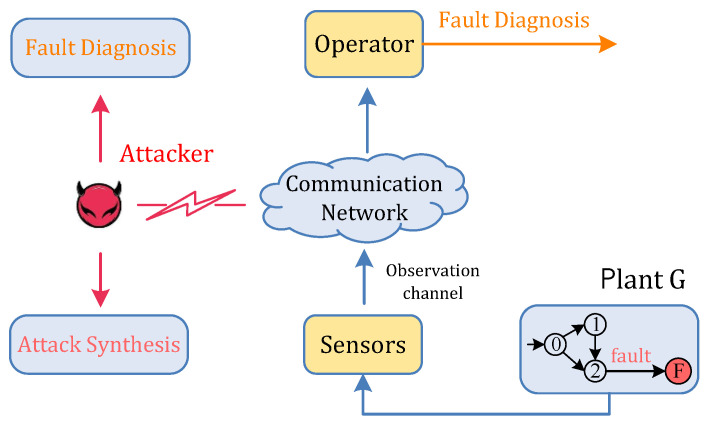
Fault diagnosis architecture under attack. To make the architecture more illustrative, different components are represented by different colors.

Motivated by this, our focus is on a specific class of cyber-attacks called sensor network attacks, which may potentially inject false network signals into the communication channels between sensors and the system operator, as shown in Figure 1. As will be clear in the context, in the worst-case scenario, due to the impact of an attacker, the operator cannot diagnose the fault occurrence as it should. Indeed, sensor network attacks have been investigated in the context of attack detection [18,19], state estimation [20,21], and supervisory control [22,23]. However, very few studies have been devoted to fault diagnosis under attack. Apart from our earlier work [24], two recent contributions in this context are [25,26]. In these works, from a defense viewpoint, the authors aim to capture the fact that fault diagnosis can be properly performed against malicious attacks.

In this paper, we study from the attacker’s viewpoint, which allows us to determine conditions under which the attacker is harmful and stealthy and synthesize a strategy to satisfy those conditions. Our objective is to verify a successful attacker that can achieve two goals: (i) induce the operator to no longer diagnose the fault occurrence, guaranteeing its harmfulness; (ii) at the same time, maintain itself as undetected by the operator, guaranteeing its stealthiness. To this end, we first propose a bipartite diagnoser called a joint diagnoser (JD) that embeds all possible attacks in a given attack scenario. To capture stealthy attacks only, a refined JD, i.e., stealthy joint diagnoser (SJD), is presented, which finally provides a necessary and sufficient condition for the existence of a successful attacker.

Another contribution of this work is the development of a successful attack synthesis algorithm. To ensure stealthiness, an attacker must consider the race between inserted events and internal system events. In essence, when synthesizing successful attacks, an attack event must be inserted at the desired time. In order to capture this race, we define a pre-empting state of the SJD as those states corresponding to the case where the internal system events are pre-empted by the inserted events. Based on this, a successful attack synthesis algorithm is finally proposed. By providing a general synthesis approach, our ultimate goal is to understand what kinds of attacks systems are not robust to and how to reduce their impact on diagnosis estimation performance. In Table 1, we compare the proposed approach with previous studies.

Compared with our conference paper [24] that introduced the diagnosis setting without providing proofs of the results, we here provide formal proofs and detailed examples. Furthermore, we present an approach for online synthesizing a successful attacker.

## 3. Preliminaries

### 3.1. Finite-State Automata

We first review some common notations of DESs used throughout this paper; the reader is referred to [6] for more details. It is assumed that a DES to be diagnosed is modeled as a finite-state automaton (automaton for short) $G=(Q,\Sigma ,\delta ,q_{0})$, where *Q* is the finite set of states, Σ is the finite set of events, δ:Q×Σ→Q is the partial transition function, and $q_{0}\in Q$ is the initial state. Suppose that set Σ is divided into the observable event set $\Sigma_{o}$ and the unobservable event set $\Sigma_{uo}$. The set of active events at state *q* is defined as Γ(q)={σ∈Σ|δ(q,σ)!}, where “!” means “defined”. The language generated by *G*, denoted by L(G) or simply *L*, is defined as $L=\{s\in \Sigma^{*}|\delta \left(q_{0},s\right)!\}$, where $\Sigma^{*}$ denotes the Kleene closure of Σ. Let *s* denote a word of *L*. The set of the prefixes of *s* is denoted as $\bar{s}=\left\{u\in \Sigma^{*}|\left(\exists v\in \Sigma^{*}\right)\left[uv=s\right]\right\}$.

The natural projection $P:\Sigma^{*}\to \Sigma_{o}^{*}$ is defined as (where ε is the empty word)

$$\;P\left(\varepsilon \right)=\varepsilon \;\;\mathrm{and}\;P\left(s\sigma \right)=\left\{\begin{array}{ccc} \;P(s)\sigma , & \mathrm{if} & \;\sigma \in \Sigma_{o};\\ \;P(s),\; & \mathrm{if} & \;\sigma \in \Sigma_{uo}. \end{array}\right.$$

The inverse projection $P^{-1}:\Sigma_{o}^{*}\to 2^{\Sigma^{*}}$ is defined as $P^{-1}\left(w\right)=\left\{s\in L\left(G\right)\;|\;P\left(s\right)=w\right\}$, i.e., $P^{-1}\left(w\right)$ consists of all words *s* in L(G) whose observations are *w*.

Let $G_{1}=\left(Q_{1},\Sigma_{1},\delta_{1},q_{01}\right)$ and $G_{2}=\left(Q_{2},\Sigma_{2},\delta_{2},q_{02}\right)$. The parallel composition between $G_{1}$ and $G_{2}$ is defined as $G_{1}\|G_{2}=Ac\left(Q_{1}\times Q_{2},\Sigma_{1}\cup \Sigma_{2},\delta ,\left(q_{01},q_{02}\right)\right)$, where $\delta \left[\left(q_{1},q_{2}\right),\sigma \right]=\left(q_{1}^{\prime },q_{2}^{\prime }\;\right)\;\mathrm{if}\;\delta_{1}\left(q_{1},\sigma \right)=\;q_{1}^{\prime }$ and $\delta_{2}\left(q_{2},\sigma \right)=q_{2}^{\prime }$; $\delta \left[\left(q_{1},q_{2}\right),\sigma \right]=\left(q_{1}^{\prime },q_{2}\right)\;\mathrm{if}\;\delta_{1}\left(q_{1},\sigma \right)=q_{1}^{\prime }$ and $\sigma \notin \Sigma_{2}$; $\delta \left[\left(q_{1},q_{2}\right),\sigma \right]=\left(q_{1},q_{2}^{\prime }\right)\;\mathrm{if}\;\delta_{2}\left(q_{2},\sigma \right)=q_{2}^{\prime }$ and $\sigma \notin \Sigma_{1}$; otherwise, it is undefined. In the definition of $G_{1}\|G_{2}$, Ac(·) denotes the accessible part of an automaton.

### 3.2. Fault Diagnosis

In the literature of fault diagnosis in DESs, the unobservable event set $\Sigma_{uo}$ is further partitioned into the set of regular unobservable events $\Sigma_{reg}$ and the set of fault events $\Sigma_{f}$. Let $\Psi \left(\Sigma_{f}\right)=\left\{sf\in L|s\in \Sigma^{*},f\in \Sigma_{f}\right\}$ denote the set of all finite words in *L* that end with a fault event *f*. With some abuse of notation, $\Sigma_{f}\in s$ denotes that $\bar{s}\cap \Psi \left(\Sigma_{f}\right)\neq \emptyset$. A word *s* is said to be faulty (resp., normal) if $\Sigma_{f}\in s$ (resp., $\Sigma_{f}\notin s$). The fault diagnosis problem in a DES aims to decide, using the current observation $w\in \Sigma_{o}^{*}$, if a fault has already occurred or not. To solve this problem, one wishes to build a diagnosis function $\gamma :\Sigma_{o}^{*}\to \left\{N,F,U\right\}$ associating each observation to a diagnosis state such that

$$\gamma \left(w\right)=\left\{\begin{array}{c} N,\;\;\;\;\;\;\mathrm{if}\;\forall s\in P^{-1}\left(w\right),\Sigma_{f}\notin s;\\ F,\;\;\;\mathrm{if}\;\forall s\in P^{-1}\left(w\right),\Sigma_{f}\in s;\\ U,\;\;\;\mathrm{otherwise}. \end{array}\right.$$

In other words, an observation *w* is called normal if γ(w)=N since, in this case, no word producing *w* contains a fault; faulty if γ(w)=F since, in this case, all words producing *w* contain a fault; ambiguous otherwise. A standard way to compute the diagnosis function is by using a diagnoser. The diagnoser of *G* is defined as

(1) $$Diag\left(G\right)=\left(Q_{d},\Sigma_{o},\delta_{d},q_{d,0}\right).$$

**Figure 2:**
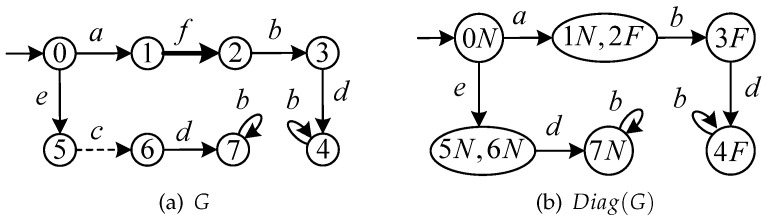
(**a**) A plant *G* and (**b**) its diagnoser Diag(G).

Its construction procedure is detailed in [6]. By construction, it holds L(Diag(G))=P(L(G)). The diagnoser state space $Q_{d}$ is a subset of $2^{Q\times \{N,F\}}$. The diagnoser allows one to associate every state to a diagnosis value $\gamma \left(q_{d}\right)=\gamma \left(w\right)$, where $q_{d}=\delta_{d}\left(q_{d,0},w\right)$. The diagnoser state $q_{d}$ is negative if $\gamma (q_{d})=N$; positive if $\gamma (q_{d})=F$; uncertain if $\gamma (q_{d})=U$. Figure 2a shows a plant *G*, where $\Sigma_{o}=\left\{a,b,d,e\right\}$, $\Sigma_{uo}=\left\{c,f\right\}$, and $\Sigma_{f}=\left\{f\right\}$. The corresponding diagnoser is shown in Figure 2b.

## 4. Sensor Network Attacks

**Figure 3:**
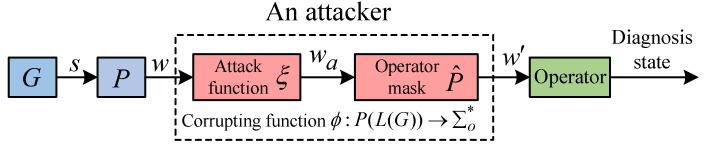
A fault diagnosis system under attack.

We start by defining a system model to be diagnosed under attack depicted in Figure 3, where the shadowed block denotes a sensor network attacker that intervenes in the communication channels between the sensors and the operator. It is assumed that the attacker can compromise a subset of the sensor network channels. Specifically, it may implement two types of sensor network attacks: (i) Sensor Erasure attack (SE-attack), which erases some readings generated by the plant; (ii) Sensor Insertion attack (SI-attack), which inserts some fake readings that have not occurred in the plant. If a plant generates a word $s\in \Sigma^{*}$, the attacker observes an original observation w=P(s) and then produces a corrupted observation $w^{\prime }$. The operator computes diagnosis state $\gamma (w^{\prime })$ from the corrupted observation $w^{\prime }$.

To formally define the attack function in Figure 3, we denote by $\Sigma_{era}\subseteq \Sigma_{o}$ (resp., $\Sigma_{ins}\subseteq \Sigma_{o}$) the set of events subject to SE-attacks (resp., SI-attacks). Correspondingly, we define $\Sigma_{-}=\left\{\sigma_{-}|\sigma \in \Sigma_{era}\right\}$ as the set of erased events and $\Sigma_{+}=\left\{\sigma_{+}|\sigma \in \Sigma_{ins}\right\}$ as the set of inserted events. We also define $\Sigma_{a}=\Sigma_{o}\cup \Sigma_{+}\cup \Sigma_{-}$ as the attack alphabet.

**Definition 1.**  Given a plant G with sets $\Sigma_{ins}$ and $\Sigma_{era}$, an attack function $\xi :P\left(L\left(G\right)\right)\to \Sigma_{a}^{*}$ satisfies the following constraints:

*(1)* $\xi \left(\varepsilon \right)\in \Sigma_{+}^{*}$;

*(2)* ∀wσ∈P(L(G)), where $w\in \Sigma_{o}^{*}$ and $\sigma \in \Sigma_{o}$:

(2) $$\xi \left(w\sigma \right)\in \left\{\begin{array}{cc} \;\xi \left(w\right)\left\{\sigma \right\}\Sigma_{+}^{*} & \mathrm{if}\;\;\sigma \in \Sigma_{o}\setminus \Sigma_{era},\\ \;\xi \left(w\right)\left\{\sigma_{-},\sigma \right\}\Sigma_{+}^{*} & \mathrm{if}\;\;\sigma \in \Sigma_{era}. \end{array}\right.$$

Statement (1) in Definition 1 implies that the attacker may insert an arbitrary word $t\in \Sigma_{+}^{*}$ at the initial state before any generated word of *G* is observed. By Statement (2), the attacker cannot erase σ if σ does not belong to $\Sigma_{era}$. However, it may insert an arbitrary word $t\in \Sigma_{+}^{*}$ after σ. If an event $\sigma \in \Sigma_{era}$ occurs, the attacker may either erase σ or leave it intact, and then insert any arbitrary word $t\in \Sigma_{+}^{*}$. Compared with the nondeterministic attack function in [22], the attack function ξ in Definition 1 is deterministic; therefore, in order to provide different classes of attack strategies, we define the set of all possible attack functions depending on relativity to sets $\Sigma_{era}$ and $\Sigma_{ins}$ as $“\Xi (\Sigma_{era},\Sigma_{ins})”$, abbreviated as Ξ.

An attack function ξ introduces an attack language, denoted as L(ξ,G)={ξ(w)|w∈P(L(G))} (abbreviated as L(ξ)). Let $w_{a}\in L\left(\xi \right)$ be an attack word. Given an attack word $w_{a}$, to describe the corrupted observation $w^{\prime }$ received by the operator, an operator mask $\hat{P}:\Sigma_{a}^{*}\to \Sigma_{o}^{*}$ is defined as

(3) $$\hat{P}\left(\varepsilon \right)=\varepsilon ,\hat{P}\left(w_{a}\sigma^{\prime }\right)=\left\{\begin{array}{c} \;\hat{P}\left(w_{a}\right)\sigma \;\mathrm{if}\;\sigma^{\prime }=\sigma \in \Sigma_{o}\vee \sigma^{\prime }=\sigma_{+}\in \Sigma_{+},\\ \;\hat{P}\left(w_{a}\right)\;\mathrm{if}\;\sigma^{\prime }=\sigma_{-}\in \Sigma_{-}. \end{array}\right.$$

We now define two diagnosis functions on $\Sigma_{a}$. The attacker diagnosis function $\gamma_{att}:\Sigma_{a}^{*}\to \left\{N,F,U\right\}$ is defined as $\gamma_{att}\left(w_{a}\right)=\gamma \left(w\right)$, where $w_{a}=\xi \left(w\right)$; the operator diagnosis function $\gamma_{opr}:\Sigma_{a}^{*}\to \left\{N,F,U\right\}$ is defined as $\gamma_{opr}\left(w_{a}\right)=\gamma \left(w^{\prime }\right)$, where $w^{\prime }=\hat{P}\left(w_{a}\right)$.

**Definition 2.**  A corrupting function $\phi :P\left(L\left(G\right)\right)\to \Sigma_{o}^{*}$ is defined as $\phi \left(w\right)=\hat{P}\left(\xi \left(w\right)\right)$, where $\xi :P\left(L\left(G\right)\right)\to \Sigma_{a}^{*}$ and $\hat{P}:\Sigma_{a}^{*}\to \Sigma_{o}^{*}$ are the attack function and the operator mask, respectively.

In this paper, a sensor network attacker is characterized by a corrupting function that takes an original observation *w* as input and produces a corrupted observation $w^{\prime }=\phi \left(w\right)=\hat{P}\left(\xi \left(w\right)\right)$ as output, as shown in Figure 3. We define $L\left(\phi ,G\right)=\hat{P}\left(L\left(\xi ,G\right)\right)$, abbreviated as L(ϕ), as the corrupted language induced by function ϕ. The set of all possible corrupting functions on $\Sigma_{era}$ and $\Sigma_{ins}$ is defined as $“\Phi (\Sigma_{era},\Sigma_{ins})”$, abbreviated as Φ.

## 5. Problem Statement

In this section, we first state the fault diagnosis problem under attack as follows.

**Problem 1.**  *(Online diagnosis under attack) Given an attack word $w_{a}\in L\left(\xi \right)$, determine if the diagnosis states based on the corresponding w and $w^{\prime }$ are consistent. *

In the worst-case scenario, a faulty observation *w* that allows for the detection of a fault (i.e., positive diagnosis state “F”) can be corrupted into a normal or ambiguous observation corresponding to the absence of the fault (i.e., negative diagnosis state “N”) or to an uncertain situation (i.e., uncertain diagnosis state “U”). This leads to the notion of a harmful attacker, defined as follows.

**Definition 3.**  *An attacker ϕ for a language L is said to be harmful if there exists an observation w∈P(L) with γ(w)={F}, which may be corrupted into another observation $w^{\prime }=\phi \left(w\right)$ and $\gamma \left(w^{\prime }\right)\in \left\{N,U\right\}$.*

Under such a harmful attacker (that receives *w*), the occurrence of a fault is hidden from the operator (that perceives $w^{\prime }$); that is, a harmful attacker introduces a delay in the detection of the fault. Next, the stealthiness of an attacker is considered. To this end, we introduce an attack detection mechanism [20,22]. If an attacker can always keep its attacks undiscovered by the operator during system execution, we call it stealthy. We now give the condition that implies the stealthiness of an attacker.

**Definition 4.**  *An attacker ϕ for a language L is stealthy if L(ϕ)⊆P(L(G)). *

The stealthiness of an attacker is ensured when any corrupted observation received by the operator is contained in the observed language of *G*. Associated with Definition 4 are two sets of words $w\in \Sigma_{a}^{*}$ defined as follows. Given a plant *G*, the set of stealthy words on $\Sigma_{a}$ is defined as $S_{s}=\left\{w_{a}\in \Sigma_{a}^{*}|\hat{P}\left(w_{a}\right)\in P\left(L\left(G\right)\right)\right\}$, while the set of exposing words on $\Sigma_{a}$ is defined as $S_{e}=\left\{w_{a}\sigma \in \Sigma_{a}^{*}|w_{a}\in S_{s},\sigma \in \Sigma_{a},w_{a}\sigma \notin S_{s}\right\}$. A stealthy word $w_{a}$ produces a corrupted observation $w^{\prime }=\hat{P}\left(w_{a}\right)\in P\left(L\left(G\right)\right)$, which does not reveal the attacker’s presence. On the contrary, an exposing word results in the exposure of the attacker at the last observable step. Finally, the ideal attacker that can achieve both goals is said to be successful. Finally, from the attack viewpoint, we formalize the successful attacker existence and synthesis problem.

**Problem 2.**  *(Existence and synthesis of a successful attacker) Given a language L, determine whether there exists a successful attacker ϕ for L. If it exists, synthesize the successful attacker ϕ. *

## 6. Stealthy Joint Diagnoser

### 6.1. Attacker Diagnoser and Operator Diagnoser

In our previous work [24], we detail the constructions of two special diagnosers, called Attacker Diagnoser and Operator Diagnoser. The former diagnoser describes all attack words $w_{a}$ that can be generated under attack. The latter diagnoser describes how all words $w_{a}\in \Sigma_{a}^{*}$ are recognized by the operator. Starting from a diagnoser $Diag\left(G\right)=\left(Q_{d},\Sigma_{o},\delta_{d},q_{d,0}\right)$, we build the following:

- Attacker Diagnoser $Diag_{att}\left(G\right)=\left(Q_{d},\Sigma_{a},\delta_{att},q_{d,0}\right)$ through self-looping each state with all events in $\Sigma_{+}$ and then adding in parallel to each event $\sigma \in \Sigma_{era}$ the corresponding event $\sigma_{-}\in \Sigma_{-}$.
- Operator Diagnoser $Diag_{opr}\left(G\right)=\left(Q_{d}\cup q_{\emptyset },\Sigma_{a},\delta_{opr},q_{d,0}\right)$ through self-looping each state with all events in $\Sigma_{-}$, then adding in parallel to each event $\sigma \in \Sigma_{ins}$ the corresponding event $\sigma_{+}\in \Sigma_{+}$, and finally adding a dump state $q_{\emptyset }$ that is reached by all undefined transitions.

**Figure 4:**
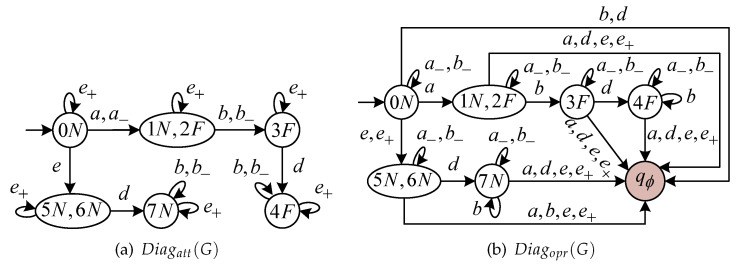
(**a**) $Diag_{att}\left(G\right)$ and (**b**) $Diag_{opr}\left(G\right)$ for *G* in Figure 2a.

**Example 1.**  *Consider the plant G in Figure 2a. Let $\Sigma_{ins}=\left\{e\right\}$ and $\Sigma_{era}=\left\{a,b\right\}$. The corresponding $Diag_{att}\left(G\right)$ and $Diag_{opr}\left(G\right)$ are shown in Figure 4a and Figure 4b, respectively. *

**Proposition 1.**  *Given a nominal diagnoser $Diag\left(G\right)=\left(Q_{d},\Sigma_{o},\delta_{d},q_{d,0}\right)$, let $Diag_{att}\left(G\right)=\left(Q_{d},\Sigma_{a},\delta_{att},q_{d,0}\right)$ be the attacker diagnoser. It holds that*

*(1) $w_{a}\in L\left(Diag_{att}\left(G\right)\right)\Leftrightarrow \left(\exists \xi \in \Xi \right)(\exists w\in L\left(Diag\left(G\right)\right)\;\left[w_{a}=\xi \left(w\right)\right]$;*

*(2) $\left(\forall \xi \in \Xi \right)\left(\forall w\in L\left(Diag\left(G\right)\right)\right)\left[\delta_{att}\left(q_{d,0},\xi \left(w\right)\right)=\delta_{d}\left(q_{d,0},w\right)\right]$.*

**Proof.**  Consider first Statement (1). (⇐) By the construction of $Diag_{att}\left(G\right)$, given a word w∈L(Diag(G)) and ξ∈Ξ, there exists a corresponding attack word $w_{a}\in L\left(Diag_{att}\left(G\right)\right)$ such that $w_{a}=\xi \left(w\right)$. (⇒) Consider a word $w_{a}\in$$L(Diag_{att}\left(G\right))$ that reaches a state $Q_{d}$ and only contains events in Σ, implying an original observation. At each state of $Diag_{att}\left(G\right)$, all events $\sigma_{+}$ are in a self-loop, which implies that $w_{a}$ can be corrupted by inserting a word of fake events in $\Sigma_{ins}$. If the word $w_{a}$ is generated by executing a transition $\delta_{att}\left(Q_{d}^{\prime },\sigma \right)=Q_{d}^{\prime \prime }$ with $\sigma \in \Sigma_{era}$, it may happen that the “parallel" transition $\delta_{att}\left(Q_{d}^{\prime },\sigma_{-}\right)=Q_{d}^{\prime \prime }$ is executed corresponding to an attack that erases σ. Therefore, there exists a function ξ∈Ξ and an observation w∈L(Diag(G)) such that $\xi \left(w\right)=w_{a}$. The proof of Statement (2) follows that of Statement (1).    □

By Statement (1) in Proposition 1, the language of the attacker diagnoser consists of all possible attack words *w*. According to Statement (2), the diagnosis state estimation of $Diag_{att}\left(G\right)$ based on *w* is the same as that of Diag(G) based on *s*, where $w_{a}=\xi \left(w\right)$.

**Proposition 2.**  *Given a nominal diagnoser $Diag\left(G\right)=\left(Q_{d},\Sigma_{o},\delta_{d},q_{d,0}\right)$, let $Diag_{opr}\left(G\right)=\left(Q_{d}\cup q_{\emptyset },\Sigma_{a},\delta_{opr},q_{d,0}\right)$ be the operator diagnoser. It holds that*

*(1) $L\left(Diag_{opr}\left(G\right)\right)=S_{s}\cup S_{e}$*;

*(2) $w_{a}\in S_{s}\subseteq L\left(Diag_{opr}\left(G\right)\right)\Rightarrow \left(\exists w^{\prime }\in L\left(Diag\left(G\right)\right)\right)\left[w^{\prime }=\hat{P}\left(w_{a}\right)\right]$;*

*(3) $\forall w_{a}\in L\left(Diag_{opr}\left(G\right)\right)$: if $w_{a}\in S_{s}$, $\delta_{opr}\left(q_{d,0},w_{a}\right)=\delta_{d}\left(q_{d,0},\hat{P}\left(w_{a}\right)\right)$; if $w_{a}\in S_{e}$, $\delta_{opr}\left(q_{d,0},w_{a}\right)=q_{\emptyset }$*.

**Proof.**  Statement (1) is first considered. By construction the $Diag_{opr}\left(G\right)$, it includes all the words in $S_{s}\cup S_{e}$. The following is to prove that all the words $w_{a}\in L\left(Diag_{opr}\left(G\right)\right)$ either belong to $S_{s}$ or $S_{e}$. Consider a word $w_{a}\in L\left(Diag_{opr}\left(G\right)\right)$ that reaches a state $q_{d}\in Q_{d}$ and only contains events in $\Sigma_{o}$, implying an original observation received by the operator. At each state, all events $\sigma_{-}$ are in a self-loop, which corresponds to the generation of $w_{a}$. By the definition of $\hat{P}$, it holds that $\hat{P}\left(w_{a}\right)\in P\left(L\left(G\right)\right)$, i.e., $w_{a}\in S_{s}$. If the word $w_{a}$ is generated by executing a transition $\delta_{opr}\left(q_{d}^{\prime },\sigma_{+}\right)=q_{d}^{\prime \prime }$ with $\sigma \in \Sigma_{ins}$, it may happen that the “parallel” transition $\delta_{att}\left(q_{d}^{\prime },\sigma \right)=q_{d}^{\prime \prime }$ is executed and thus $\hat{P}\left(w_{a}\right)\in P\left(L\left(G\right)\right)$, i.e., $w\in S_{s}$. Then, if the word $w_{a}$ yields $d_{\emptyset }$, then $\hat{P}\left(w_{a}\right)\notin P\left(L\left(G\right)\right)$, i.e., $w_{a}\in S_{e}$, which completes the proof of Statement (1). The proofs of Statement (2) and (3) follow that of Statement (1).    □

By Statement (1) in Proposition 2, all words in $S_{s}$ and $S_{e}$ can be generated by $Diag_{opr}\left(G\right)$. By Statement (2), a word in $S_{s}$ generated by $Diag_{opr}\left(G\right)$ can correspond to a corrupted observation $w^{\prime }\in P\left(L\left(G\right)\right)$ recognized by the operator. Statement (3) implies that (i) the diagnosis state estimation of $Diag_{opr}\left(G\right)$ based on a stealthy word $w_{a}\in S_{s}$ is the same as that of Diag(G) based on $w^{\prime }$, where $w^{\prime }=\hat{P}\left(w_{a}\right)$; (ii) all exposing words $w_{a}\in S_{e}$ yield $q_{\emptyset }$.

### 6.2. Joint Diagnoser and Its Refining

**Definition 5.**  *A joint diagnoser (JD for short) J-Diag(G) is defined as J-$Diag\left(G\right)=\left(X,\Sigma_{a},\delta_{a},x_{0}\right)$$=Diag_{att}\left(G\right)$$∥Diag_{opr}\left(G\right)$. *

As defined, every state of *J*-Diag(G) is a pair $x=(q_{d},{\bar{q}}_{d})$. The states of *J*-Diag(G) can be partitioned into stealthy states and exposing states as follows.

**Definition 6.**  *Given a joint diagnoser J-$Diag\left(G\right)=\left(X,\Sigma_{a},\delta_{a},x_{0}\right)$, the set of exposing states is defined as $X_{e}=\left\{x=\left(q_{d},{\bar{q}}_{d}\right)\in X\;|\;{\bar{q}}_{d}=q_{\emptyset }\right\}$; the set of stealthy states is defined as $X_{s}=X\setminus X_{e}$. *

If an exposing word $w_{a}\in S_{e}$ yields an exposing state, we conclude that the attacker that produces $w_{a}$ is stealthy. However, if a stealthy word $w_{a}\in S_{s}$ yields a stealthy state, it can only be inferred that the attacker producing $w_{a}$ is currently undiscovered. There is a case where, following the future evolution of *G*, the attacker will be inevitably discovered. This leads to the notion of a weakly exposing region, denoted as $X_{we}⊇X_{e}$, which can be computed iteratively by a procedure in [21]. In the first iteration,

(4) $$\begin{array}{cc} \; & X_{we}:=\left\{x\in X|\left(\exists \sigma \in \Sigma_{o}\right)\left[\delta_{a}\left(x,\sigma \right)\in X_{e}\Rightarrow \sigma \notin \Sigma_{era}\wedge \left(\forall \sigma^{\prime }\in \Sigma_{ins}\right)\left[\delta_{a}\left(x,\sigma^{\prime }\right)\in X_{e}\right]\right]\right\}. \end{array}$$

The remaining iterations are executed similarly to Equation (4). We do not present the complete procedure here for the sake of brevity but illustrate it via Example 2. Dually, we define the strongly stealthy region as $X_{ss}=X\setminus X_{we}\subseteq X_{s}$.

**Figure 5:**
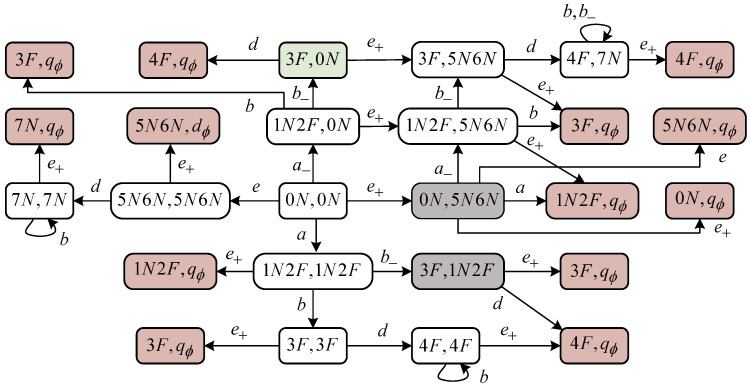
*J*-Diag(G) in Example 2.

**Example 2.**  *The joint diagnoser for the preceding G is shown in Figure 5, where the exposing states in $X_{we}$ are highlighted in brown while the stealthy states in $X_{we}$ are highlighted in gray. Let us focus on a stealthy state ({3F},{1N,2F}). In the case that the occurrence of event d will take ({3F},{1N,2F}) to an exposing state $\left(\left\{4F\right\},\left\{q_{\emptyset }\right\}\right)$, we find that i) d cannot be erased; ii) the inserted event $e\in \Sigma_{ins}$ will reach another exposing state $\left(\left\{3F\right\},\left\{q_{\emptyset }\right\}\right)$. By Equation (4), $\left(\left\{3F\right\},\left\{1N,2F\right\}\right)\in X_{we}$ holds. In plain words, once such a stealthy state is reached, all attempts of an attacker to prevent it from reaching a subsequent exposing state will fail. *

**Definition 7.**  *The stealthy joint diagnoser (SJD, for short) with respect to a joint diagnoser J-Diag(G)=$\left(X,\Sigma_{a},\delta_{a},x_{0}\right)$ is defined as SJ-$Diag\left(G\right)=Ac\left(X_{ss},\Sigma_{a},\delta_{sa},x_{0}\right)$, where $X_{ss}=X\setminus X_{we}$ and $\delta_{sa}={\left.\delta_{a}\right|}_{X_{ss}\times \Sigma_{a}\to X_{ss}}$. *

The resulting SJD is obtained by removing all states in $X_{we}$ from a JD and taking its accessible part. Then, we define a subset of the states of SJ-Diag(G), called pre-empting states, which will be used in the procedure to extract an attack function from SJ-Diag(G).

**Definition 8.**  *Given a JD J-Diag(G)=$\left(X,\Sigma_{a},\delta_{a},\;x_{0}\right)$ with the corresponding SJD SJ-$Diag\left(G\right)\;=\;\left(X_{ss},\;\Sigma_{a},\;\delta_{sa},\;x_{0}\right)$, the set of pre-empting states of SJ-Diag(G) is defined as*

$$X_{p}=\left\{x_{ss}\in X_{ss}\;|\;\left(\exists \sigma \in \Sigma_{o}\right)\;\left[\delta_{a}\left(x_{ss},\sigma \right)\in X\setminus X_{ss}\;\wedge \;\left(\sigma \notin \Sigma_{era}\vee \delta_{a}\left(x_{ss},\sigma_{-}\right)\in X\setminus X_{ss}\right)\right]\right\};$$

*the set of non-pre-empting states of SJ-Diag(G) is defined as $X_{np}=X_{ss}\setminus X_{p}$. *

A state $x_{ss}\in X_{ss}$ is pre-empting if i) there exists an event $\sigma \in \Sigma_{o}$ whose occurrence (even if erased) causes *J*-Diag(G) to lead outside $X_{ss}$; ii) however, σ can be definitely pre-empted by inserting an appropriate word of events in $\Sigma_{+}$ to reach a state in the strongly stealthy state. This state captures the races between inserted events and internal system events. Specifically, at a pre-empting state, the inserted word must be performed before the execution of event σ. In Example 2, state ({3F},{0N}) is a pre-empting state (highlighted in green), where event *d* must be pre-empted by inserting a fake event $e_{+}$ to reach a state $\left(\left\{3F\right\},\left\{5N,6N\right\}\right)\in X_{ss}$; otherwise, the occurrence of $d\in \Sigma_{o}\setminus \Sigma_{era}$ will take ({3F},{0N}) to an exposing state $\left(\left\{4F\right\},\left\{d_{\emptyset }\right\}\right)$.

### 6.3. Synthesis of Attackers

In order to synthesize an attacker, the following definition is first required.

**Definition 9.**  *Let $x_{ss}\in X_{ss}$ be a state and $X_{np}$ be the set of non-pre-empting states in the SJD SJ-$Diag\left(G\right)=\left(X_{ss},\Sigma_{a},\delta_{sa},x_{0}\right)$.*

*(1) The set of events in $\Sigma_{a}$ enabled at $x_{ss}$ is defined as $\Gamma_{SJ}\left(x_{ss}\right)=\left\{\sigma \in \Sigma_{a}\;|\;\delta_{sa}\left(x_{ss},\sigma \right)!\right\}$.*

(2) The set of words consisting of events in $\Sigma_{+}$ that originate from $x_{ss}$ and lead to a non-pre-empting state is defined as $L_{+}(SJ$-$Diag\left(G\right),x_{ss})=\left\{w_{a,+}\in \Sigma_{+}^{*}\;|\;\delta_{sa}\left(x_{ss},w_{a,+}\right)\in X_{np}\right\}$. 

The following objective is to show how an attacker may determine an attack function from an SJD. In [21], an algorithmic procedure is presented for this purpose. The main idea is to select attack words that do not end in any state that has an inserted event as a successor transition, of course, including the pre-empting state. Compared with [21], it is now expected to synthesize the attack function producing an attack word that only does not end in pre-empting states. This means that an attacker cannot stay in the pre-empting state when determining its attack function. Now, we provide the following attacker synthesis algorithm, i.e., Algorithm 1.
**Algorithm 1** Synthesis of the corrupting function ϕ**Require: **$G=(Q,\Sigma ,\delta ,q_{0})$ and SJ-$Diag\left(G\right)=(X_{ss},\Sigma_{a},\delta_{sa},$$x_{0})$

**Ensure:** corrupting function ϕ∈Φ
1:ϕ←Synth(SJD)2:**procedure** Synth(SJD)3:      initialization: w←ε4:      select a word $w_{a,+}\in L_{+}(SJ$-$Diag\left(G\right),x_{0})$5:      $\xi \left(w\right):=w_{a,+}$; $\phi \left(w\right):=\hat{P}\left(\xi \left(w\right)\right)$6:      $x_{ss}:=\delta_{sa}\left(x_{0},w_{a,+}\right)$7:      **while** a new observable event $\sigma \in \Sigma_{o}$ is produced by *G* **do**8:           I:=∅9:           **if** $\sigma \in \Gamma_{SJ}\left(x_{ss}\right)$ **then** I:=I∪{σ}10:         **if** $\sigma_{-}\in \Gamma_{SJ}\left(x_{ss}\right)$ **then** $I:=I\cup \{\sigma_{-}\}$11:         select an event $\sigma^{\prime }\in I$ and a word $w_{a,+}\in L_{+}(SJ$-$Diag\left(G\right),\delta_{sa}\left(x_{ss},\sigma^{\prime }\right))$12:         let $w_{a}:=\sigma^{\prime }w_{a,+}$ and $w_{a}^{\prime }\in \left\{\sigma^{\prime },w_{a}\right\}$13:         let $x_{ss}:=\delta_{sa}\left(x_{ss},\sigma^{\prime }\right)$14:         **if** $x_{ss}\in X_{np}$ **then** $\xi \left(w\sigma \right)\in \xi \left(w\right)w_{a}^{\prime }$ and $x_{ss}:=\delta_{sa}\left(x_{ss},w_{a}^{\prime }\right)$15:         **if** $x_{ss}\in X_{p}$ **then** $\xi \left(w\sigma \right):=\xi \left(w\right)w_{a}$ and $x_{ss}:=\delta_{sa}\left(x_{ss},w_{a}\right)$16:         $\phi \left(w\sigma \right):=\hat{P}\left(\xi \left(w\sigma \right)\right)$17:         w←wσ18:    **end while**19:**end procedure**


This algorithm may recursively associate each observation generated by *G* with a corresponding attack word. We now briefly explain how it works. When no event is generated by *G*, the attacker may insert a suitable word $w_{a,+}$ in the set $L_{+}(SJ$-$Diag\left(G\right),x_{0})$ (Steps 3 and 4). In Step 5, ξ(ε) is computed, and the new current state $x_{ss}$ of SJ-Diag(G) is updated. Then, we wait for *G* to generate a new observable event σ (Step 7). A new set *I* initialized at the empty set is defined. If σ is enabled at $x_{ss}$, σ is added to *I*. If $\sigma_{-}$ is enabled at $x_{ss}$, $\sigma_{-}$ is added to *I* (Steps 8 to 10). In Step 11, an event $\sigma^{\prime }\in I$ and the insertion of a word $w_{a,+}\in L_{+}(SJ$-$Diag\left(G\right),\delta_{sa}\left(x_{ss},\sigma^{\prime }\right))$ are selected. If the current state $x_{ss}$ with the occurrence of $\sigma^{\prime }$ is a non-pre-empting state, an attack word $w_{a}^{\prime }$ is produced, which is either $\sigma^{\prime }$ or the concatenation of $\sigma^{\prime }$ and $w_{a,+}\in L_{+}(SJ$-$Diag\left(G\right),\delta_{sa}\left(x_{ss},\sigma^{\prime }\right))$ (Step 14). Otherwise, another attack word $w_{a}$ is produced as the concatenation of $\sigma^{\prime }$ and $w_{a,+}$ (Step 15). The corresponding corrupting function is computed in Step 16. Step 17 updates the observation *w* to wσ. This procedure goes to Step 7 when a new observable event is generated by *G*.

**Proposition 3.**  *Given a plant G, function ϕ is stealthy if and only if, for all w∈P(L(G)), the corrupted observation ϕ(w) can be computed by Algorithm 1.*

According to the property of the SJD, the above results trivially hold, which means that the corrupting function synthesized by Algorithm 1 must be stealthy. Using the notion of pre-empting states, we provide a characterization of the SJD as follows.

**Theorem 1.**  *Given a plant G, let Diag(G)$=(Q_{d},\Sigma_{o},\delta_{d},q_{d,0})$ be the diagnoser and SJ-Diag(G)$=(X_{ss},\Sigma_{a},\delta_{sa},x_{0})$ be the SJD. The following implication holds:*

$$\begin{array}{cc} \; & \;\left(\exists \;\xi \left(w\right)\in \Sigma_{a}^{*}\right)\;\;\left[\delta_{sa}\left(x_{0},\xi \left(w\right)\right)=x_{ss}=\left(q_{d},{\bar{q}}_{d}\right)\wedge x_{ss}\in X_{np}\right]\\ \; & \;\Leftrightarrow (\exists w\in P(L(G))),(\exists \;\xi \;\;\mathrm{synthesized}\;\mathrm{by}\;\mathrm{Algorithm}\;1)\\ & \;\;\;\;\;\;\;\;\;[q_{d}=\delta_{d}\left(q_{d,0},w\right)\;\;\wedge \;\;{\bar{q}}_{d}=\delta_{d}\left(q_{d,0},\hat{P}\left(\xi \left(w\right)\right)\right)=\delta_{d}\left(q_{d,0},\phi \left(w\right)\right)]. \end{array}$$

**Proof.**  (If) Assume that there exists an observation w∈P(L(G)) and an attack function ξ such that $q_{d}=\delta_{d}\left(q_{d,0},w\right)$ and ${\bar{q}}_{d}=\delta_{d}\left(q_{d,0},\hat{P}\left(\xi \left(w\right)\right)\right)$, where the attack function ξ is synthesized by Algorithm 1. By Proposition 3, the attack word ξ(w) is a stealthy word, i.e., $\xi \left(w\right)\in S_{s}$. By Propositions 1 and 2, we have $\delta_{att}\left(q_{d,0},w_{a}\right)=\delta_{d}\left(q_{d,0},w\right)$ and $\delta_{opr}\left(q_{d,0},w_{a}\right)=\delta_{d}\left(q_{d,0},\hat{P}\left(w_{a}\right)\right)$, i.e., $q_{d}=\delta_{att}\left(q_{d,0},w_{a}\right)$ and ${\bar{q}}_{d}=\delta_{opr}\left(q_{d,0},w_{a}\right)$. By *J*-$Diag\left(G\right)=Diag_{att}\left(G\right)$$∥Diag_{opr}\left(G\right)$, and by the definition of SJ-Diag(G), we have $\delta_{sa}\left(x_{0},w_{a}\right)=x_{ss}=\left(q_{d},{\bar{q}}_{d}\right)$. Since ξ(w) is computed by selecting $w_{a}$, which ends in $x_{ss}$, by Algorithm 1, $x_{ss}\in X_{np}$.

(Only if) Assume that there exists an attack word $\xi \left(w\right)\in \Sigma_{a}^{*}$ in SJ-Diag(G) such that $\delta_{sa}\left(x_{0},\xi \left(w\right)\right)=x_{ss}=\left(q_{d},{\bar{q}}_{d}\right)$ and $x_{ss}\in X_{np}$. By *J*-$Diag\left(G\right)=Diag_{att}\left(G\right)$$∥Diag_{opr}\left(G\right)$, and by the definition of SJ-Diag(G), it holds that $\delta_{att}\left(q_{d,0},\xi \left(w\right)\right)=q_{d}$ and $\delta_{opr}\left(q_{d,0},\xi \left(w\right)\right)={\bar{q}}_{d}$. By Propositions 1 and 2, we have $\delta_{att}\left(q_{d,0},\xi \left(w\right)\right)=\delta_{d}\left(q_{d,0},w\right)$ and $\delta_{opr}\left(q_{d,0},\xi \left(w\right)\right)=\delta_{d}\left(q_{d,0},\hat{P}\left(\xi \left(w\right)\right)\right)$, i.e., $q_{d}=\delta_{d}\left(q_{d,0},w\right)$ and ${\bar{q}}_{d}=\delta_{d}\left(q_{d,0},\hat{P}\left(\xi \left(w\right)\right)\right)$.    □

According to the above result, a state pair $x_{ss}=\left(q_{d},{\bar{q}}_{d}\right)$ in the joint diagnoser reached by an attack word $w_{a}$ represents the joint diagnosis state estimation, where $q_{d}$ describes the original diagnosis state estimation of the attacker based on the original observation *w*, where $w_{a}=\xi \left(w\right)$, and ${\bar{q}}_{d}$ describes the corrupted diagnosis state estimation of the operator based on the corrupted observation $w^{\prime }=\hat{P}\left(w_{a}\right)$.

Finally, we conclude this section by the complexity analysis of the proposed approach. Let Diag(G) be a nominal diagnoser with $|Q_{d}|$ states. By construction, the attacker diagnoser $Diag_{att}\left(G\right)$ has the same set of states of Diag(G), and so does the operator diagnoser $Diag_{opr}\left(G\right)$ except for the dump state $d_{\emptyset }$. Since the JD *J*-Diag(G) is the parallel composition of $Diag_{att}\left(G\right)$ and $Diag_{att}\left(G\right)$, its maximum number of states is $|Q_{d}\left|\times \right|Q_{d}+1|$. Hence, the complexity of building an SJD is $O(|Q_{d}{|}^{2})$, which is polynomial in the number of states of the nominal diagnoser. However, it is well known that the construction of the diagnoser is worst-case exponential in the number of states in the system. Hence, the overall computational complexity of an SJD is exponential in the number of states of *G*.

## 7. Fault Diagnoisis under Attack

We first address Problem 1, i.e., online fault diagnosis under attack. Similar to the nominal setting, we begin with the definition of a diagnosis function pair.

**Definition 10.**  Given an attack word $w_{a}$, a diagnosis pair function $r:\Sigma_{a}^{*}\to \left\{N,F,U\right\}\times \left\{N,F,U\right\}$ associating to $w_{a}\in \Sigma_{a}^{*}$ a diagnosis state pair is defined as $r\left(w_{a}\right)=\left(\gamma_{att}\left(w_{a}\right),\gamma_{opr}\left(w_{a}\right)\right)$, where $\gamma_{att}$ and $\gamma_{opr}$ are the attacker and operator diagnosis functions, respectively. 

From the definitions of $\gamma_{att}$ and $\gamma_{opr}$, it holds that $r\left(w_{a}\right)=\left(\gamma_{att}\left(w_{a}\right),\gamma_{opr}\left(w_{a}\right)\right)=\left(\gamma \left(w\right),\gamma \left(w^{\prime }\right)\right)$, where $w_{a}=\xi \left(w\right)$ and $w^{\prime }=\hat{P}\left(w_{a}\right)$. A more systematic way to compute the diagnosis pair function is by using an SJD. Let $x_{ss}=\left(q_{d},{\bar{q}}_{d}\right)=\delta_{sa}\left(x_{0},w_{a}\right)$. By Theorem 1, the SJD allows one to associate every state to a diagnosis state pair $r\left(x_{ss}\right)=r\left(w_{a}\right)$, i.e., $\gamma \left(q_{d}\right)=\gamma \left(w\right)$ and $\gamma \left({\bar{q}}_{d}\right)=\gamma \left(w^{\prime }\right)$ are the diagnosis state of the attacker and operator, respectively. Therefore, Problem 1 can be solved by tracking the current state in SJD reached by the attack word $w_{a}$. The set of all diagnosis state pairs is defined as R={N,F,U}×{N,F,U}, which can be partitioned into $R=R_{c}\cup R_{w}\cup R_{h}$, where $R_{c}=\left\{\left(N,N\right),\left(U,U\right),\left(F,F\right)\right\}$, $R_{w}=\left\{\left(N,U\right),\left(N,F\right),\left(U,N\right),\left(U,F\right)\right\}$, and $R_{h}=\left\{\left(F,N\right),\left(F,U\right)\right\}$.

**Definition 11.**  *Let SJ-Diag(G) be an SJD. A state $x_{ss}$ is correct if $r\left(x_{ss}\right)\in R_{c}$; wrong non-harmful if $r\left(x_{ss}\right)\in R_{w}$; harmful if $r\left(x_{ss}\right)\in R_{h}$. Denote the set of correct states, the set of wrong non-harmful states, and the set of harmful states by $X_{sc}$, $X_{sw}$, and $X_{sh}$, respectively. *

When the SJD is in a correct state, the operator correctly computes the diagnosis state regardless of the fact that an attack has occurred. When the SJD reaches a wrong non-harmful state, the operator computes a wrong diagnosis state from the corrupted observation due to an attack, which is inconsistent with the diagnosis state based on the original observation. Note that, in such a case, the fault diagnosis is manipulated due to the attack but does not pose a harmful danger. For example, there exists a special case where an attacker induces the operator to think that a fault has occurred, while the system is actually under nominal behavior. The false positive may make the operator decide to take some unnecessary actions, e.g., shut down the system and start it again, which may increase the operational expenses of operating a system.

Finally, harmful states of the SJD correspond to the detection of a fault based on the original observation, while no detection is based on corrupted observation. Its physical interpretation is that the attacker itself has already confirmed that the fault has occurred, but it induces the operator to be unable to claim the fault occurrence.

### 7.1. Verification of Successful Attackers

As discussed above, it is intuitive that the presence of a harmful state in the SJD is related to Problem 2, the existence of a successful attacker.

**Theorem 2.**  *Given a plant G, there exists a successful attacker if and only if the SJD SJ-Diag(G) contains a harmful state, i.e., $X_{sh}\neq \emptyset$.*

**Proof.**  (If) Suppose that SJ-Diag(G) contains a harmful state $x_{ss}$ such that $r\left(x_{ss}\right)\in R_{h}$, where $x_{ss}=\delta_{sa}\left(x_{0},w_{a}\right)$. By Theorem 1, associated with $x_{ss}$ is an attack word $w_{a}$ such that $r\left(w_{a}\right)=r\left(x_{ss}\right)\in R_{h}$. Hence, there exists an attacker ϕ that alters the observation *w* into $w^{\prime }$ such that $\left(\gamma \left(w\right),\gamma \left(w^{\prime }\right)\right)=\left(\gamma_{att}\left(w_{a}\right),\gamma_{opr}\left(w_{a}\right)\right)=r\left(w_{a}\right)$, i.e., $\left(\gamma \left(w\right),\gamma \left(w^{\prime }\right)\right)\in \left\{\left(F,N\right),\left(F,U\right)\right\}$. By Definition 3, the attacker ϕ is harmful. By the construction of SJ-Diag(G), ϕ must satisfy stealthiness. Hence, the attacker ϕ is successful.

(Only if) Suppose that there exists a successful attacker ϕ. From Definition 3, there exists an observation *w* such that *w* is corrupted into $w^{\prime }$ satisfying $\left(\gamma \left(w\right),\gamma \left(w^{\prime }\right)\right)\in \left\{\left(F,N\right),\left(F,U\right)\right\}$. By Theorem 1, there exists a reachable state $x_{ss}$ in SJ-Diag(G) with the occurrence of $w_{a}$ such that $r\left(x_{ss}\right)=\left(\gamma \left(q_{d}\right),\gamma \left({\bar{q}}_{d}\right)\right)=\left(\gamma \left(w\right),\gamma \left(w^{\prime }\right)\right)\in \left\{\left(F,N\right),\left(F,U\right)\right\}$.    □

**Figure 6:**
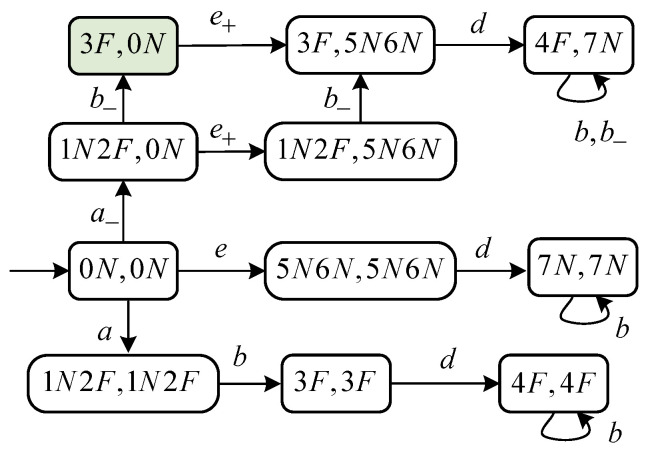
SJ-Diag(G) in Example 3.

**Example 3.**  *Let us continue with Example 2. After refining, the SJD is shown in Figure 6. Let $w_{a,1}=a_{-}e_{+}$ be an attack word that yields the wrong non-harmful state ({1N,2F},{5N,6N}). This implies that the diagnosis state of the attacker based on w=a is “U” while the diagnosis state of the operator based on $w^{\prime }=e$ is “N”. At this point, the attacker has doubted if the fault has occurred or not; however, the operator is certain that the fault has not occurred.*

*Let the evolution continue. Another word $w_{a,2}=a_{-}e_{+}b_{-}d$ yields a harmful state ({4F},{7N}) in SJ-Diag(G). By Theorem 2, there exists a successful attacker for G that produces the attack word $w_{a,2}=a_{-}e_{+}b_{-}d$. At this point, the attacker is certain that the fault has occurred based on w=abd; however, based on the corrupted observation $w^{\prime }=ed$, the operator persists in its opinion that the fault has not occurred. *

### 7.2. Synthesis of Successful Attackers

We first provide the following proposition to ensure that a successful attacker can be synthesized from the SJD.

**Proposition 4.**  *Let G be a plant and SJ-$Diag\left(G\right)=(X_{ss},\Sigma_{a},$$\delta_{sa},x_{0})$ be the SJD. A successful corrupting function ϕ can be computed if and only if $X_{sh}\cap X_{np}\neq \emptyset$.*

**Proof.**  (If) Suppose that a state $x_{ss}\in X_{sh}\cap X_{np}$ in SJ-Diag(G) satisfies $x_{ss}=\delta_{sa}\left(x_{0},w_{a}\right)$ and $w_{a}=\xi \left(w\right)$, where w∈P(L(G)). By $x_{ss}\in X_{np}$, the attack word $w_{a}$ does not end in a pre-empting state. According to Algorithm 1 and Proposition 3, we see that ϕ is stealthy. Since $x_{ss}=\left(q_{d},{\bar{q}}_{d}\right)\in X_{sh}$, by Theorem 1, we have $x_{ss}=\delta_{sa}\left(x_{0},w_{a}\right)=\left(q_{d},{\bar{q}}_{d}\right)$ such that $\delta_{d}\left(q_{d,0},w\right)=q_{d}$ and $\delta_{d}\left(q_{d,0},w^{\prime }\right)={\bar{q}}_{d}$ with $w^{\prime }=\phi \left(w\right)$, where the attacker ϕ alters the observation *w* into $w^{\prime }$ such that $\left(\gamma \left(w\right),\gamma \left(w^{\prime }\right)\right)=\left(\gamma_{att}\left(w_{a}\right),\gamma_{opr}\left(w_{a}\right)\right)=r\left(w_{a}\right)=r\left(x_{ss}\right)\in R_{h}$, i.e., $\left(\gamma \left(w\right),\gamma \left(w^{\prime }\right)\right)\in \left\{\left(F,N\right),\left(F,U\right)\right\}$. According to Definition 3, the corrupting function ϕ is also harmful.

(Only if) Suppose that ϕ is a successful corrupting function that can be computed. Since function ϕ is stealthy, by Algorithm 1 and Proposition 3, the attack word $w_{a}=\xi \left(w\right)$ does not end in a pre-empting state of SJ-Diag(G), i.e., $x_{ss}=\delta_{sa}\left(x_{0},\xi \left(w\right)\right)\in X_{np}$. On the other hand, as function ϕ is harmful, according to Definition 3, there exists an observation w∈P(L(G)) that can be changed into an observation $w^{\prime }=\phi \left(w\right)\in P\left(L\left(G\right)\right)$, and $\left(\gamma \left(w\right),\gamma \left(w^{\prime }\right)\right)\in \left\{\left(F,N\right),\left(F,U\right)\right\}$. By $w,w^{\prime }\in P\left(L\left(G\right)\right)$, it holds that $\delta_{d}\left(q_{d,0},w\right)=q_{d}$ and $\delta_{d}\left(q_{d,0},w^{\prime }\right)={\bar{q}}_{d}$. Since SJ-Diag(G) contains all possible stealthy attacks, by Theorem 1, there must exist a state $x_{ss}=\left(q_{d},{\bar{q}}_{d}\right)$ in SJ-Diag(G) with the occurrence of $w_{a}=\xi \left(w\right)$ such that $\delta_{d}\left(q_{d,0},w\right)=q_{d}$ and $\delta_{d}\left(q_{d,0},w^{\prime }\right)={\bar{q}}_{d}$. By $r\left(x_{ss}\right)=r\left(w_{a}\right)=\left(\gamma_{att}\left(w_{a}\right),\gamma_{opr}\left(w_{a}\right)\right)=\left(\gamma \left(w\right),\gamma \left(w^{\prime }\right)\right)\in \left\{\left(F,N\right),\left(F,U\right)\right\}$, i.e., $r\left(x_{ss}\right)\in R_{h}$, we see that the state $r_{ss}$ is harmful. It holds that $x_{ss}\in X_{sh}\cap X_{np}$, i.e., $X_{sh}\cap X_{np}\neq \emptyset$.    □

This proposition implies that, from the synthesis viewpoint, only a successful attacker that determines an attack word yielding a non-pre-empting harmful state can make sense. We now give Algorithm 2 to synthesize a successful attacker from an SJD. In Algorithm 2, the condition $x_{ss}\in X_{sh}\cap X_{np}$ (Step 3) for selecting a state *r* guarantees the harmfulness of the stealthy corrupting function synthesized by Algorithm 1.
**Algorithm 2** Synthesis of the successful corrupting function ϕ**Require:** $G=(Q,\Sigma ,\delta ,q_{0})$ and SJ-$Diag\left(G\right)=(X_{ss},\Sigma_{a},\delta_{sa},$$x_{0})$ **Ensure:** successful corrupting function ϕ
1:ϕ←Synthsuc(SJD)2:**procedure** Synthsuc(SJD)3:     **if** $X_{sh}\cap X_{np}$ **then** choose a harmful state $x_{ss}\in X_{sh}\cap X_{np}$ with $x_{ss}=\delta_{sa}\left(x_{0},w_{a}\right)$4:     compute the observation *w* such that $w_{a}=\xi \left(w\right)$5:     **while** there is an observation $w^{*}\in \bar{w}\cap P\left(L\left(G\right)\right)$ **do**6:           compute the corrupted observation $\phi (w^{*})$ by Algorithm 17:     **end while**8:**end procedure**

**Example 4.**  Following Algorithm 2, choose a harmful state $\left(4F,7N\right)\in X_{sh}\cap X_{np}$. Associated with the state is an attack word $w_{a}=a_{-}b_{-}e_{+}d\in L(SJ$-Diag(G)). It corresponds to an observation w=abd. For all $w^{*}\in \bar{w}\cap P\left(L\left(G\right)\right)$, the attack function ξ is computed by Algorithm 1—ξ(ε)=ε, $\xi \left(a\right)=a_{-}$, $\xi \left(ab\right)=a_{-}b_{-}e_{+}$, and $\xi \left(abd\right)=a_{-}b_{-}e_{+}d$—and the corrupting function is synthesized accordingly: ϕ(ε)=ε, ϕ(a)=ε, ϕ(ab)=e, and ϕ(abd)=ed. The key point is that the attack word $a_{-}b_{-}$ is not selected in the synthesis procedure, i.e., it holds that $\xi \left(ab\right)=a_{-}b_{-}e_{+}$ rather than $\xi \left(ab\right)=a_{-}b_{-}$. This means that the attacker implements its attacks by first erasing the occurrence of event a, then erasing event b, and finally inserting $e_{+}$ while observing abd.

## 8. Conclusions and Future Work

This paper investigated the problem of fault diagnosis under sensor network attacks. The notion of a successful attacker is proposed; it can degrade fault diagnosis without being discovered by the operator. We construct a joint diagnoser (JD) to describe all the behavior of the system under all possible sensor attacks. Then, the SD is refined to stealthy joint diagnoser (SJD), which includes stealthy attacks only. Based on it, a necessary and sufficient condition for the existence of a successful attacker is presented. Finally, we propose an algorithmic procedure to synthesize successful attacks from the SJD. In the future, we plan to extend the proposed diagnoser-based approach to diagnosability verification under attack. On the other hand, the decentralized setting should be further considered.

## Figures and Tables

**Table 1:** Comparison of related works, where the "✓" symbol indicates the selection of an option.

Reference	Sensor Failures	Sensor Attacks	Attack Detection	Fault Diagnosis	Attack Synthesis
[15,16,17]	✓			✓	
[18,19]		✓	✓		
[20,21,22,23]		✓	✓		✓
[25,26]		✓		✓	
This work		✓	✓	✓	✓

## Data Availability

Enquiries about data availability should be directed to the authors.

## Abbreviations

The following abbreviations are used in this manuscript:

DES	Discrete event system
CPS	Cyber–physical system
JD	Joint diagnoser
SJD	Stealthy joint diagnoser
*G*	Plant automaton
Diag(G)	Diagnoser
$Diag_{att}\left(G\right)$	Attacker diagnoser
$Diag_{opr}\left(G\right)$	Operator diagnoser
γ	Diagnosis function
$\gamma_{att}$	Attacker diagnosis function
$\gamma_{opr}$	Operator diagnosis function
*r*	Diagnosis pair function
*P*	Natural projection
ξ	Attack function
$\hat{P}$	Operator mask
